# Fatigue-related symptom clusters and functional status of older adults in hospice

**DOI:** 10.1017/S1478951522000207

**Published:** 2023-02

**Authors:** Suzan F. Abduljawad, Jason W. Beckstead, Debra Dobbs, Constance Visovsky, Carmen S. Rodriguez, Susan C. McMillan, Hsiao Lan Wang

**Affiliations:** 1 Lombardi Comprehensive Cancer Center, Georgetown University Medical Center, Washington, DC;; 2 College of Public Health, University of South Florida, Tampa, FL;; 3 School of Aging Studies, University of South Florida, Tampa, FL;; 4 College of Nursing, University of South Florida, Tampa, FL;; 5 Emeritus Distinguished Professor, University of South Florida, Tampa, FL; 6 School of Nursing, The University of Alabama at Birmingham, Birmingham, AL

**Keywords:** Depression, Elderly, Functionality, Lack energy, Symptom co-occurrence

## Abstract

**Background.:**

Fatigue is frequently co-existing with other symptoms and is highly prevalent among patients with cancer and geriatric population. There was a lack of knowledge that focus on fatigue clusters in older adults with cancer in hospice care.

**Objectives.:**

To identify fatigue-related symptom clusters in older adult hospice patients and discover to what extent fatigue-related symptom clusters predict functional status while controlling for depression.

**Method.:**

This was a cross-sectional study in a sample of 519 older adult hospice patients with cancer, who completed the Memorial Symptom Assessment Scale, the Center for Epidemiological Studies Depression, Boston Short Form Scale, and the Palliative Performance Scale. Data from a multi-center symptom trial were extracted for this secondary analysis using exploratory factor analysis and hierarchical multiple regression analysis.

**Results.:**

Data from 519 patients (78 ± 7 years) with terminal cancer who received hospice care under home healthcare services revealed that 39% of the participants experienced fatigue-related symptom clusters (lack of energy, feeling drowsy, and lack of appetite). The fatigue cluster was significantly associated positively with depression (*r* = 0.253, *p* < 0.01), and negatively with functional status (*r* = −0.117, *p* < 0.01) and was a strong predictor of participants’ low functional status. Furthermore, depression made a significant contribution to this predictive relationship.

**Conclusion.:**

Older adult hospice patients with cancer experienced various concurrent symptoms. The fatigue-specific symptom cluster was identified significantly associated with depression and predicted functional status. Fatigue should be routinely monitored in older adults, especially among hospice cancer patients, to help reduce psychological distress and prevent functional decline.

## Background

By 2030, it is predicted that 22.1 million Americans will be living with cancer, and almost two-thirds (64%) of this population will be 65 years or older (Miller et al., 2019). There are multiple issues in the symptom research among older adults (Hernandez Torres and Hsu, 2017). First, aging is often heterogeneous, and chronological age does not reflect biological age (Loh et al., 2020). Treatment-limiting frailty, cancer-related cognitive decline (Pergolotti et al., 2020), other age-related concerns such as slowed metabolism, and multi-morbidities keep older patients from participating in clinical trials (Todd et al., 2021; VanderWalde et al., 2021), and subject them to experiencing a rapidly progressive course of illness and increased disability. Hence, there are detrimental effects on their well-being, mortality, and survival (Kirkhus et al., 2019; Pergolotti et al., 2020; Sedrak et al., 2020). Symptom management is one of the most crucial components in their comprehensive hospice care. Those patients frequently experience a complex of distressing and co-occurring symptoms, as well as higher levels of fatigue than their younger counterparts (Yates et al., 2015; Soones et al., 2021).

Symptom cluster refers to the presence of a group of related co-occurring symptoms which can have an adverse effect on patients’ health outcomes and may also have a combined effect as a predictor of patient’s morbidity (Miaskowski et al., 2007). Patients with cancer who are in hospice care and who were treated with surgery, radiation, or chemotherapy often experience multiple symptoms that occur simultaneously (Omran et al., 2017). Depression, sleep disturbances, pain, poor appetite, and difficulty concentrating are symptoms that often occur in a cluster with fatigue (Dong et al., 2016; Nieder and Kämpe, 2017; Loh et al., 2018). To date, intervention research that focuses on managing fatigue symptom cluster is scarce, and mostly targets patients actively receiving cancer therapy or cancer survivors (So et al., 2020; Li, 2021). Therefore, there is a need to understand fatigue symptom clusters and to further improve future designs of fatigue cluster intervention research in older adult hospice population.

The estimated prevalence of cancer-related fatigue in older adults with cancer is 70% (Soones et al., 2021). Fatigue in older adults with cancer is a risk factor for functional disabilities, some of these individuals experience heightened levels of fatigue, performing their routine daily activities becomes more difficult and challenging (Williams et al., 2021). Often, family or friends are the ones who provide most of the support, the higher the functional impairment the more is the caregiver burden, such dependency can have a negative influence on the elderly’s role identity related to a perceived loss of control, switched roles in the family, when adult children become the caregivers of their parents, and worsening of quality of life (Esbensen et al., 2012), with an increased risk for healthcare system utilization through hospitalization (Nightingale et al., 2021).

Functional status is defined as the ability to conduct daily living activities that are executive in nature and necessary for self-care independence, such as cooking, feeding, taking prescribed medications, bathing, housekeeping, transportation, and money management to name a few (Overcash, 2015). There is a pressing need to examine fatigue-related symptom clusters, as these clusters can be associated with a decline in functional status among older adult hospice patients (Dong et al., 2016; Reich et al., 2017). Understanding fatigue symptom clusters and their relationship with functional status in older adults with cancer can help in developing home services that enhance patient and family care, treatment strategies, and can help identify the educational requirements of patients and their caregivers.

Depression also has a significant influence on functional status, and it affects the ability to care for oneself and can predict the poor quality of life (Overcash, 2015; Grotmol et al., 2017). It is also a component of the geriatric syndromes in older adults with cancer, with a prevalence reaching 43% in palliative care patients (Kozlov et al., 2019). A striking resemblance of major depression symptoms, including loss of energy, sleep disturbance, anorexia, fatigue, and weight loss, overlaps with cancer-related symptoms often experienced by older adults (Francoeur, 2014; Soones et al., 2021). Thus, there is a need to statistically control for depression to understand fatigue-related symptom cluster’s relationships with functional status, without the influence from depression. The area where the unique contribution was needed in the symptom cluster science is to understand fatigue-related symptom clusters in high-risk populations among older adult cancer patients under hospice care.

### Research aims

We carried out this secondary analysis of data from a multisite randomized clinical trial (R01/5R01NR008252), and our aims were:

Aim 1: To identify fatigue-related symptom clusters in older adult hospice patients.

Aim 2: To examine to what extent fatigue-related symptom clusters predict functional status while controlling for depression in older adult hospice patients.

## Methods

### Sample and setting

We used data from a National Institutes of Health funded randomized controlled trial study, conducted in 2011 among newly admitted adult patients (age ≥ 18) with advanced cancer in two hospices in the Southwest of Florida (*N* = 709), who received standardized baseline symptom assessment under home care services within 24–72 h of admission. The inclusion criteria for participation were identified by admission face sheets. Eligible participants were able to read and understand English, able to pass screening with the Short Portable Mental Status Questionnaire for cognitive competency with 8 or more correct answers out of 10 (Pfeiffer, 1975). Patients were excluded if they were actively dying, comatose, excessively debilitated, or confused.

To be included in this secondary data analysis, the participants had to be older adults, who met the following criteria: age ≥ 65 (*n* = 533), completed the Palliative Performance Scale (PPS), Center for Epidemiological Studies Depression, Boston Short Form (CESD-10), and the Memorial Symptom Assessment Scale (MSAS), as well as demographic and clinical survey on admission to the study.

### Procedures

The study protocol was approved by the university’s Institutional Review Board (IRB). Participants provided written consent, and the procedures for the parent study are reported elsewhere (McMillan et al., 2012). The principal investigator of the parent study assisted with de-identified data extraction. The data set was cleaned and re-organized by removing outliers and missing data (2.6%). Missing data occurred when no data value was stored for MSAS or depression variables, and were managed with pairwise deletion techniques, assuming that data were missing completely at random. After data cleaning and eligibility criteria screening, 519 cases were available for data analyses.

### Measures

#### Sociodemographic and clinical data

Sociodemographic data included age, gender, ethnic background, education level, marital status, living arrangement, cancer type, and length of time since diagnosis in years. Living arrangement had seven items including: “living alone, living with a spouse/partner, living with spouse/partner and children, living with children (no spouse/partner), living with roommate (not spouse/partner), and living with others.” Clinical data included cancer diagnosis and the number of years from diagnosis.

#### Functional status

PPS scores were used to assess the functional status of persons receiving palliative care (Anderson et al., 1996). It was proposed to provide a framework for measuring the progressive decline in palliative care patients. The PPS measures three areas: mobility, intake, and level of consciousness in five sub-categories (the ability to perform physical activities; extent of disease; ability to do self-care; degree of ambulation; food and fluid intake; and state of consciousness). The PPS yields a single score ranging from 0 to 100% at 10% increments, with a high score indicating high-performance level. The strong positive correlations between PPS and Karnofsky Functional Status (*r* = 0.88–0.97) support its construct validity in a palliative care population. The Inter-rater reliability was strong (*r* = 0.95) in hospice population (McMillan et al., 2012).

#### Symptom clusters

The original MSAS measures the prevalence, intensity, and distress of 32 symptoms that are commonly associated with cancer therapy (Portenoy et al., 1994). A short form of MSAS was used for this study, which was revised for hospice patients with cancer, retaining items from the original MSAS that were most relevant to hospice patients’ symptom experience (McMillan et al., 2012). In addition to fatigue, the shortened MSAS measures difficulty concentrating, pain, cough, feeling nervous, dry mouth, nausea, vomiting, feeling drowsy, numbness/tingling in hands and feet, difficulty sleeping, feeling bloated, problems with urination, shortness of breath, diarrhea, feeling sad, sweats, worrying, problems with sex, itching, lack of appetite, dizziness, difficulty swallowing, feeling irritable, and constipation. The revised MSAS measures a total of 25 physical and psychological symptoms experienced in the past 7 days. Each symptom occurrence was rated 0 (“No”) or 1 (“Yes”). If their answer was yes, they were further asked about that symptom severity and distress. Symptom severity and distress subscales were rated from 0 to 4 each using a 5-point Likert-type scale. The revised MSAS has well-documented validity and reliability for use with cancer patients receiving hospice home care (McMillan and Small, 2002). For this secondary data analysis, the fatigue item was used and operationally defined as “lack of energy” in this scale.

#### Depression

Depressive symptoms were assessed using the Center for Epidemiological Studies Depression, Boston Short Form (CESD-10) Scale. The CESD-10 is a 10-item, self-report questionnaire that was developed to measure symptoms of depression in community populations. It is rated on a dichotomous 0 (“No”) to 1 (“Yes”) scale (Kohout et al., 1993). CESD-10 scores range from 0 to 10; higher scores indicate higher depressive symptoms. It combines ease of administration and reduced questionnaire burden with only 2 min of administration time. It has been utilized successfully in the assessment of depressive symptoms in cancer research (Carpenter et al., 1998; Hann et al., 1999; Garrison et al., 2011; Stagl et al., 2015). The correlation between the short form and the full CES-D was 0.88, supporting the construct validity of the CESD-10. Cronbach’s *α* was 0.92, and test–retest reliability was 0.83 for this short form (Irwin et al., 1999).

### Data analyses

Descriptive statistics including frequencies, percentages, means, and standard deviations were utilized to depict the sociodemographic and clinical data of the study population, using IBM SPSS Statistics for Windows software version 25.0 (Chicago, IL). SPSS was also used to compute scales’ scores and reliability testing, examine correlations among study variables, and conduct factor analysis. Factor analysis is often used to examine patterns of variations, and correlation among responses to the scale’s items that represent the construct being measured.

First, we used the bivariate Pearson correlation coefficient to explore the strength of associations among the 25-symptoms on the MSAS scale including fatigue, and to identify which symptoms’ correlations with fatigue reached statistical significance. MSAS symptom occurrence scores were clustered by exploratory factor analysis (EFA) to identify symptom clustering patterns among the variables. To assess sampling adequacy for factor analysis, we used the Kaiser–Meyer–Olkin (KMO) test >0.6 and Bartlett’s test of sphericity. We applied the principal component analysis (PCA) extraction method with oblique rotations (direct Oblimin), as we hypothesized to have non-zero correlations among the factors and aimed to make inferences about the relationship between physical and psychological symptoms; orthogonal rotations were inappropriate. The number of factors extracted was determined using the eigenvalue 1.0, a scree plot, and parallel analysis with the Monte Carlo data simulation technique.

Next, bivariate correlations were conducted among fatigue-related symptom clusters, depression (CESD-10), and functional status (PPS). Then, hierarchical multiple regressions with a functional status variable as the outcome variable were applied. In the first step, the predictor variable was the fatigue-related symptom cluster identified by correlations. In the second step, the predictor variable was the fatigue-related symptom cluster controlling for age, gender, and living arrangement. In the third step, the predictor variable was fatigue-related symptom cluster controlling for age, gender, living arrangement, and depression.

## Results

### Sample demographic and clinical characteristics

The sample consisted of 519 hospice patients with cancer. The average age of the participants was 78 years; over 50% male, predominantly European ancestry (96%), and 64% currently married had good social support from spouse/significant other or friend; most common cancer types were lung (36.2%) and pancreatic (9.4%) and mean time since diagnosis was 38 months. Fatigue (86.9), pain (69.9), and sleeping difficulty (38.9) were the most frequently reported symptoms. On average, patients reported experiencing 9.63 (SD = 4.19) concurrent symptoms. The average depression score was 2.89 (SD = 2.2). The average functional status PPS score was 56.83 (SD = 10.72). Patients’ characteristics are presented in Table 1.

### Research Aim 1: To identify fatigue-related symptom clusters in older adult hospice patients

#### Identifying fatigue cluster

Fatigue had significant positive correlations with the following items in relationship strength order: lack of appetite (*r* = 0.267, *p* <05), feeling drowsy (*r* = 0.220, *p* < 0.05), lack of concentration (*r* = 0.164, *p* < 0.05), shortness of breath (*r* = 0.132, *p* < 0.05), dizziness (*r* = 128, *p* < 0.05), feeling sad (*r* = 0.127, *p* < 0.05), feeling irritable (*r* = 0.126, *p* < 0.05), nausea (*r* = 0.122, *p* < 0.05), dry mouth (*r* = 0.117, *p* < 0.05), feeling nervous (*r* = 0.115, *p* < 0.05), problems with sex (*r* = 0.101, *p* < 0.05), problems with urination (*r* = 0.097, *p* < 0.05), and sweats (*r* = 0.091, *p* < 0.05). Lack of energy had a weak but significant positive correlation with depression (*r* = 0.217, *p* < 0.01) (Table 2).

The fatigue-related symptom cluster identified in this analysis included lack of energy, feeling drowsy, and lack of appetite. In the total of 519 participants, there were 201 (39%) who reported lack of energy, feeling drowsy, and lack of appetite simultaneously.

#### Factor analysis of symptom clusters

This cluster was further examined by conducting EFA to check if these three symptoms clustered under one factor. Initially, the data suitability for the cluster analysis was examined. MSAS symptom occurrence for this sample (*N* = 519) was reliable. The average inter-item Cronbach’s *α* coefficient of reliability was 0.735. The determinant of the correlation matrix approached zero (0.076). The KMO Index of Sampling Adequacy was 0.761. Finally, Bartlett’s test of sphericity was significant (*χ*^2^ = 1318.530, df = 231, *p* < 0.001). Based on these criteria, the inter-item correlation matrix was deemed adequate for factor analysis. The eigenvalue and the scree plot suggested four components, and the parallel analysis indicated one component. A four-component solution was chosen with 40.837% of the variance explained, and the symptom cluster PCA factor structure with the solution is presented in Table 3.

There were six items that loaded on Factor 1 (feeling sad, feeling irritable, worrying, difficulty concentrating, problem with sex, and feeling nervous), which explained 15.470% of the factor’s variance. Cronbach’s *α* coefficient for this cluster was 0.63. This factor was labeled “psychological.”

Four items loaded on Factor 2 (pain, nausea, vomiting, and sweats) and explained 7.058% of the factor’s variance. Cronbach’s *α* coefficient for this cluster was 0.607. Factor 2 was labeled “pain, gastrointestinal.”

Nine items loaded on Factor 3 (lack of energy, lack of appetite, dry mouth, constipation, feeling bloated, difficulty sleeping, feeling drowsy, dizziness, and problems with urination), and these explained 6.007% of the factor’s variance. Cronbach’s *α* coefficient for this cluster was 0.472. Factor 3 was labeled “somatic.”

Three items loaded on Factor 4 (shortness of breath, cough, and difficulty swallowing) which explained 5.451% of the factor’s variance. Cronbach’s *α* coefficient for this cluster was 0.444. Factor 4 was labeled “dyspnea, dysphagia.” Symptom clusters factor correlation matrix is presented in Table 4.

### Research Aim 2: To examine to which extent fatigue-related symptom clusters predict functional status while controlling for depression

#### Bivariate correlation

As a first step, the bivariate correlation of study variables with the fatigue symptom cluster were analyzed. The bivariate correlations matrix is presented in Table 2. The fatigue symptom cluster had a significant negative correlation with functional status (*r* = −0.117, *p* = 0.008). Fatigue symptom cluster had a positive significant correlation with depression (*r* = 0.253, *p* < 0.000). Depression had a negative significant correlation with functional status (*r* = −0.096, *p* = 0.027). On average, older adult hospice patients experienced 9 (SD = 4.1) concurrent symptoms out of 30 MSAS symptom occurrence subscales (Table 2), and the prevalence of fatigue was 86.9%. Approximately 34% of the patients reported feeling depressed, and 37.6% felt sad.

#### Predictors of functional status

Hierarchical multiple regression was performed to investigate the ability of the fatigue-related symptom cluster (fatigue, feeling drowsy, and lack of appetite) to predict functional status, after controlling for depressive symptoms. The first step of the hierarchical regression conducted with the independent variable fatigue-related symptom cluster regressed onto functional status explained 1.2% of the total variance of the PPS scores (*R*^2^ = 0.012, *F*(1,515) = 7.113, *p* = 0.008). The second step was to control for confounding variables that correlate with functional status; for this, hierarchical regression was conducted controlling for age, gender, and living arrangement. In the resulting second model, the fatigue-related symptom cluster significantly predicted functional status accounting for 4.5% of the variance (*R*^2^ = 0.045, *F* (8,508) = 0.176, *p* = 0.000). In the third step, to control for depression, the CESD-10 total scores were entered as a covariate variable, in addition to the fatigue-related symptom cluster, along with age, gender, and living arrangement variables. The third model increased the functional status predictability of the fatigue-related symptom cluster by 3.5 points (*R*^2^ Δ = 0.047–0.012 = 0.035), which was a small but a significant change (*F*Δ = 4.983, df = 1,507, *p* < 0.05). Hierarchical multiple regression analysis results are presented on Table 5.

## Discussion

Unlike previous symptom cluster research that identified one or multiple clusters using the exploratory approach, the current study used PCA to finalize the distinct fatigue-related symptom cluster in older adult hospice patients with cancer. In the order of strength of statistical relation, fatigue was clustered strongly with lack of appetite followed by drowsiness. Other symptoms that loaded on the same factor included dry mouth, constipation, bloating, difficulty sleeping, dizziness, and problems with urination; these symptoms had weak correlations with fatigue.

Previous studies have reported a similar clustering pattern of fatigue, drowsiness, and lack of appetite (Van Lancker et al., 2016; Yennurajalinikgam et al., 2016; Rha and Lee, 2017). However, the difference between the current study from previous symptom cluster studies might be a result of various factors such as cancer diagnoses, demographics, or disease trajectory. Typically, divergent and unique sets of symptoms are expected across those study populations (Kwekkeboom, 2016).

A study with advanced cancer patients (lung, breast, colorectal, and stomach) who were undergoing palliative chemotherapy found a cognitive symptom cluster which consisted of fatigue, drowsiness, and difficulty concentrating (Rha and Lee, 2017). Our study did not find the difficulty of concentration clustering with fatigue (lack of energy). The main study (McMillan et al., 2012) used the Short Portable Mental Status Questionnaire for cognitive competency as a cognition screener. It was possible that our participants had better cognition while receiving hospice care than the population undergoing palliative chemotherapy in Rha and Lee’s study.

Another study found fatigue, drowsiness, and loss of appetite clustered with pain and nausea, in adults ≥18 years, with an average age = 60, who were more ethnically diverse, with advanced cancer receiving palliative chemotherapy (Yennurajalinikgam et al., 2016). Our sample of older adults (average age = 78, predominantly non-Hispanic White ethnicity) consisted of more lung cancer cases and was not receiving chemotherapy. Whether or not chemotherapy exaggerated pain and nausea symptoms in this population is a question that needs further investigation. In addition, we applied PFA to confirm the identified cluster, which might be a more rigorous procedure when compared with the Yennurajalinikgam et al. (2016) study.

Moreover, a study from Belgium of older adults with advanced cancer who were receiving palliative chemotherapy (38%), reported physical fatigue, was clustered with lack of energy, lack of appetite, and dry mouth (Van Lancker et al., 2016). It is possible that the heterogeneity of patient’s cancer type, chemotherapy status, history of radiotherapy, and whether the patients were in an early phase of palliative therapy, or more near the end of life, present unique symptoms to the pattern matrix, lending a change in variance and composition of symptom clusters (Hsu et al., 2017; Klasson et al., 2021).

Interestedly, after controlling for depression, the fatigue-related symptom cluster identified in our study only included physical symptoms. Sleep disturbances are highly prevalent (40%) among older adult patients with cancer (Loh et al., 2018; Harrold et al., 2020). Correlations have been reported between fatigue and symptoms of sleep disturbances such as drowsiness and daytime sleepiness. Sleep disturbances often (49%) present as a part of a multi-symptom cluster (Loh et al., 2018), which was reported to negatively impact physical functioning (Harrold et al., 2020). In addition, alterations in appetite are frequent among patients with cancer with the lack of appetite resulting in malnutrition and energy loss. Especially when combined with lower body weight, alterations in appetite contribute to fatigue and demonstrate a link to role function decline (Pilgrim et al., 2015; Barajas-Galindo et al., 2017).

The identified fatigue symptom cluster (lack of energy, appetite, and feeling drowsy) has a clinically and statistically significant predictive relationship with functional status, which was validated in our Hierarchical Regression analysis. The increase in total variance explained by the fatigue-related cluster further supports the independent negative association of the fatigue symptom cluster with functional status.

Also, a trend was observed in the patients’ age and living arrangement differences (Table 5), suggesting that younger patients who lived with children may report more fatigue-related symptoms, higher depression, and lower functional status. One could infer that living with adult children may involve living with grandchildren and married children and may imply greater dependency on others. Conversely, living alone presents its own set of challenges, such as needing a caregiver. A similar linkage of physical and psychological symptoms is evident in symptom science literature, which found that fatigue, pain, and anxiety were tremendously increased in those undergoing chemotherapy with little to no social support (Kwekkeboom, 2016).

It is suggested that symptom experience begins with the occurrence; that is when the perception of a change is first noted. However, the actual symptom experience encompasses the process of evaluation and response (Linder, 2010). The judgment of the physical symptom severity, frequency, and location evokes the subjective psychological, physiological, or behavioral response. The symptom assessment scale must capture the whole experience of feelings, thoughts, and behaviors related to the symptom (Linder, 2010). Perhaps, the reason fatigue was not clustered with pain, sadness, or sleep disturbance which can be attributed to a limitation in the way fatigue was operationally defined, relying on lack of energy symptom occurrence only.

The focus of this study, the fatigue-related symptom cluster, is a latent variable that is not easy to measure directly. EFA made it possible to examine the observed co-occurring symptoms experienced by older adult hospice patients with cancer and discover the data’s underlying structure; that is, which symptoms group together to form the fatigue cluster. One of the strengths of this study is that it depicts a symptom cluster that is unique to this age group. Identifying the fatigue symptom cluster of lack of energy, drowsiness, and lack of appetite forms the basis for subsequent intervention research studies.

### Study limitations

Although symptom control and improving QOL are essential components of comprehensive hospice care, this study did not measure QOL outcomes, as the goal was to focus on exploring the relationship between fatigue symptom cluster and functional status. As an inherent limitation of secondary data analysis, the prevalence data of patients meeting specific diagnostic criteria for a sleep disorder was unavailable. Also, access to the participants’ nutritional status data was unattainable. Furthermore, other significant variables — socioeconomic status, psychotropic/opioid drug use, loss of appetite, poor physical function — experiences during this period, and the severity of diseases were not considered in this secondary data analysis, which may bias our results and conclusions. Another limitation of this study is that we used MSAS frequency of occurrence sub-scale data only, excluding symptom distress which perhaps diminishes the multidimensional nature and experience of the symptoms. These results may be different from findings of other research and should be interpreted with caution.

### Clinical nursing implications

Cancer care providers must be directed to manage cancer fatigue by considering it as a part of symptom clusters and embracing evidence in their practice. Fatigue has been likely underreported among advanced cancer patients (Klasson et al., 2021). There are very limited pharmacological treatments recommended for fatigue among the hospice care population (Mücke et al., 2015). The best evidence-based management for fatigue is exercise, yoga, mindfulness practice, and cognitive behavioral therapy (Berger et al., 2015). Therefore, clinicians may need to routinely assess fatigue among advanced cancer patients with or without hospice care. Those symptoms clustered with fatigue included drowsiness and lack of appetite; therefore, it is of importance to hospice and palliative care nurses while planning for fatigue management to consider proper management of sleep disturbance (i.e., drowsiness) and dietary intake to avoid malnutrition due to lack of appetite. It is not surprising that functional status had a significant relation to the fatigue cluster. The survivorship care providers may need to closely monitor for the decline of functional status among older adult patients with cancer, especially during hospice care. A necessary referral to rehabilitation medicine may be suggested for any future impairment or injury.

### Research implications

Current research indicates significant gaps that may be considered for further investigations. Statistical and scientific derivations for assessing symptom clusters and their predictive impacts on the functional status of hospice patients can provide methodical guidance in symptom management. Emphasis on the psychosocial impacts is needed as are innovative interventions to ameliorate symptom cluster management. Biological underpinnings should be further investigated on how they impact predictive symptom clusters and ethnic, cultural implications of psychosocial symptoms experienced. Future research ideally would utilize measuring scales that capture the full dimension of the fatigue experience and include baseline assessments of sleep disorders and nutritional status when considering fatigue symptom management.

## Conclusions

In this study, we were able to identify a fatigue-specific symptom cluster that older adult hospice patients experience. The fatigue symptom cluster that contained lack of energy, drowsiness, and lack of appetite was significantly and positively associated with the patients’ depressive symptoms. Experiencing the identified fatigue symptom cluster predicted a decline in functional status for those patients.

## Figures and Tables

**Table 1.** Demographics and clinical characteristics of patients

	Frequency	Percent	Mean	SD
Age			78.18	7.351
Gender				
Male	300	57.8		
Female	219	42.2		
Marital status				
Never married	18	3.5		
Currently married	332	64.0		
Separated	4	.8		
Divorced	42	8.1		
Widowed	122	23.5		
Ethnicity				
Caucasian	503	96.9		
African American	8	1.5		
Hispanic	5	1.0		
Asian/pacific islander	1	.2		
Other	2	.4		
Years of formal education			12.6	3.12
Cancer diagnosis				
Lung	188	36.2		
Pancreas	49	9.4		
Colon	39	7.5		
Prostate	32	6.2		
Breast	23	4.4		
Other	329	36.8		
Years since diagnosis			2.24	4.15
Living arrangement				
Alone	39	7.5		
Spouse/partner	342	65.9		
Spouse/partner and children	19	3.7		
Children (no spouse/ partner)	46	8.9		
Roommate (no spouse/ partner)	5	1.0		
Other	68	13.1		
Cancer symptoms				
Fatigue	451	86.9		
Pain	363	69.9		
Difficulty sleeping	202	38.9		
Depression (CESD-10)			2.89	2.2
Functional status (PPS)			56.83	10.72

**Table 2.** Bivariate correlations matrix of study variables with fatigue symptom cluster (*N* = 519)

	Age	Gender	PPS	CESD −10	FSC	MSAS severity	MSAS distress	MSAS occurrence	Living arrangement	Mean	SD
Age	1									78.18	7.34
Gender	−0.063	1								–	–
PPS	−0.036	−0.066	1							57.05	10.72
CESD-10	−0.045	0.021	−0.096*	1						2.84	2.15
FSC	−0.171**	−0.005	−0.117**	0.253**	1					2.07	0.90
MSAS severity	−0.181**	0.017	−0.114**	0.443**	0.548**	1				20.49	11.14
MSAS distress	−0.179**	0.025	−0.103*	0.451**	0.479**	0.887**	1			19.72	13.43
MSAS occurrence	−0.206**	−0.007	−0.046	0.421**	0.617**	0.882**	0.820**	1		9.65	4.10
Living arrange	−0.002	0.251**	0.153**	−0.045	−0.103*	−0.026	−0.052	−0.028	1	–	–

PPS, Palliative Performance Scale; functional status measure; CESD-10, Center for Epidemiological Studies Depression, Boston Short Form; FSC, fatigue-related symptom cluster; MSAS, Memorial Symptom Assessment Scale.

* Correlation is significant at the 0.01 level (2-tailed).

** Correlation is significant at the 0.05 level (2-tailed).

**Table 3:** Symptom clusters structure matrix

	Factors			
Symptom experiences	Factor 1	Factor 2	Factor 3	Factor 4
Worrying	0.688			
Feeling sad	0.684			
Feeling irritable	0.612			
Feeling nervous	0.580			
Difficulty concentrating	0.532			
Problem with sex	0.337			
Nausea		0.817		
Vomiting		0.785		
Pain		0.542		
Sweats		0.412		
Constipation			0.500	
Lack of appetite			0.489	
Feeling bloated			0.483	
Difficulty sleeping			0.476	
Dry mouth			0.460	
Fatigue; lack of energy			0.455	
Feeling drowsy			0.417	
Problems with urination			0.370	
Dizziness			0.341	
Shortness of breath				0.726
Cough				0.703
Difficulty swallowing				0.459
Variance explained	15.470%	7.058%	6.007%	5.451%
Total variance explained	33.987%			

**Table 4:** Symptom clusters factor correlation matrix

	Factor 1	Factor 2	Factor 3	Factor 4
Factor 1	1.000			
Factor 2	0.278	1.000		
Factor 3	0.324	0.270	1.000	
Factor 4	0.235	0.168	0.229	1.000

Factor 1 = “psychological”; Factor 2 = “pain, gastro-intestinal”; Factor 3 = “somatic”; Factor 4 = dyspnea, throat. Extraction Method: Principal Component Analysis. Rotation Method: Promax with Kaiser Normalization.

**Table 5.** Hierarchical multiple regression predicting functional status

Model		*B*	Std. Error	*β*	*t*	Sig.	Adjusted *R*^2^	Std. Error of the estimate	*R*^2^ change
Step 1	(Constant)	59.50	1.169		50.877	0.000	0.012	10.755	0.014
	FSC	−1.38	0.520	−0.117	−2.667	0.008			
Step 2	(Constant)	67.39	5.482		12.294	0.000	0.045	10.575	0.046
	FSC	−1.406	0.527	−0.118	−2.668	0.008			
	Living other	6.188	1.464	0.193	4.227	0.000			
	Age	−0.098	0.066	−0.067	−1.479	0.140			
	Gender	−2.489	1.034	−0.114	−2.407	0.016			
Step 3	(Constant)	67.844	5.483		12.374	0.000	0.047	10.561	0.004
	FSC	−1.209	0.542	−0.102	−2.232	0.026			
	Living other	6.164	1.462	0.193	4.216	0.000			
	Age	−0.097	0.066	−0.066	−1.460	0.145			
	Gender	−2.425	1.033	−0.111	−2.347	0.019			
	CESD-10 Total	−0.336	0.220	−0.068	−1.524	0.128			

Dependent variable: PPS total.

PPS, Palliative Performance Scale; CESD-10, Center for Epidemiological Studies Depression, Boston Short Form; FSC, fatigue-related symptom cluster.
